# Could Milkability Parameters Serve as a Reliable Tool to Predict the Morphology of Teat Structures and Their Milking-Induced Changes?

**DOI:** 10.3390/ani14243610

**Published:** 2024-12-14

**Authors:** Iveta Szencziová, Matúš Gašparík, Jaromír Ducháček, Eva Tóthová Tarová, Melinda Nagy, Luděk Stádník, Mária Mičiaková, Radim Codl

**Affiliations:** 1 Department of Biology, Faculty of Education, J. Selye University in Komárno, Bratislavská cesta 3322, 945 01 Komárno, Slovakia; tothovatarovae@ujs.sk (E.T.T.); nagym@ujs.sk (M.N.); 2 Department of Animal Science, Faculty of Agrobiology, Food and Natural Resources, Czech University of Life Sciences Prague, Kamýcká 129, 165 00 Prague, Czech Republic; gasparikm@af.czu.cz (M.G.); duchacek@af.czu.cz (J.D.); stadnik@af.czu.cz (L.S.); codl@af.cuz.cz (R.C.); 3 Institute of Animal Husbandry, Slovak University of Agriculture in Nitra, Trieda Andreja Hlinku 2, 949 76 Nitra, Slovakia; maria.miciakova@uniag.sk

**Keywords:** dairy cows, milkability, teat canal, udder health, ultrasonography, precision agriculture

## Abstract

The study aimed to explore if milkability parameters could reliably predict the dimensions of teat structures and their milking-induced changes. Ultrasonography repeatedly measured the teat structures of 48 Holstein cows from mid to late lactation. We found that milking-induced changes in each structure are affected by different milkability parameters. Regression models for teat canal change and length change were significant, and variability was found to be 46.03% and 21.50%, respectively. Similarly, the teat structure’s dimensions significantly affected milkability parameters, which differed for each structure. However, regression models only explained between 3.36% (teat length) and 7.59% (cistern) of variability. The prediction potential, performed based on milkability, is limited if the initial dimensions of structures are not provided. If teat dimensions were measured at the beginning of the production life, automatically collected milkability data could be used to calculate milking-induced changes incurred with each milking and the development of teat dimensions over the production life. If perfected, this tool could provide alerts about critical milking-induced changes and risky teat conformation traits, as they have a proven effect on udder health and are reliable indicators of milking effectiveness.

## Simple Summary

Teat morphology, milking-induced changes in teat structures, and the connection of these factors to milking parlor settings, milkability, and udder health have been heavily researched and discussed over recent decades. Ultrasonography is an invaluable tool in research; however, its practical application on the farm is limited. Nevertheless, the information it could provide about teat conformation and milking-induced changes would be useful in practice thanks to the close relationship of these factors with udder health and milking effectiveness. This study aims to test if it is possible to predict teat morphology and milking-induced changes based on milkability, which can be automatically assessed, and teat structures, which can easily be measured by ultrasonography. We found that some factors like teat length and canal and barrel thickness are significantly affected by multiple milkability parameters and have the potential to be predicted reliably. The prediction of the teat apex, wall, and cistern might be more challenging, as there is a need to include other effects. Precisely identifying these relations might help to develop practical tools that can be used on dairy farms, and further research into this topic could create a detailed computer model for changes in teat structures during milking and throughout production life without the need for ultrasonography.

## 1. Introduction

Maintaining a healthy mammary gland in dairy cows is indispensable in terms of ensuring good production performance and the animal’s well-being. The inner morphology of the lactating bovine teat and its modifications during the milking procedure play an important role in the defense against mastitis [1]. Since udder health is still one of the major reasons for culling in current dairy farming systems [2], it is important to pay special attention to it. Previous studies have already shown that the shape of the udder and teats [3,4,5], the microbiota of the teat skin and inner mucosa [5,6], and the forces applied during machine milking [7,8] influence the teat defense mechanisms and can therefore affect the mammary gland’s susceptibility to mastitis. There is general agreement that machine milking can result in congestion and edema of the teat tissue, especially at the teat end, and that it also influences the teat diameter and penetrability of the teat canal. The functional effect of impaired teat fluid circulation may be divided into two parts: teat canal closure and the passage of pathogens, and possible effects on the immunological defense mechanisms regarding antigenic detection and the initiation of immunological responses [9].

The extent of changes in the inner teat traits due to the milking process depends on the machine settings, such as the time needed to turn the machine on, the applied vacuum level, the type of teatcup liner, the mouthpiece chamber vacuum, the pulsation ratio and rate, and the milking duration [9,10,11]. Other milkability parameters were also found to affect milking-induced changes in teat structures significantly or, conversely, were affected by their morphology. Studies found that teat traits like teat canal length and width can influence milk flow [1,12,13]. Moreover, teat length and barrel thickness are important due to the proper fit of the teat into the liner, stopping its function of properly massaging the teat during milking from being impaired [14]. Gašparík et al. [15] found that desirable traits like high milk yield, short lag time, and high milk flow were associated with greater milking-induced changes in teat length. The anatomical and functional characteristics of teats can partly explain the milk flow characteristics of individual quarters [16]. Nørstebø et al. [17] concluded that the quarter milk flow rate was useful for assessing associations between milking machine function and teat-end condition; low average milk flow rates were associated with a higher likelihood of the teat having a thickened or roughened teat-end callosity ring.

Ultrasound is used for the investigation of teats’ inner morphologies, and various traits are known to be useful for measurements in ultrasonographic studies of the bovine teat [13,18,19,20,21]. However, most studies perform indirect estimates of values such as teat-end firmness, teat-end width, teat wall thickness, and teat canal length. Machine milking-induced short-term changes have been found to be associated with the risk of new intramammary infections. As measured with a modified spring-loaded caliper, an increase in machine milking-induced teat-end thickness of more than 5% significantly increased teat canal colonization [22] and the risk of new infections [23]. Furthermore, Zwertvaegher et al. [8] observed that the teats that thickened during milking had higher somatic cell counts than those that decreased in barrel diameter. Studies also found a relationship between barrel thickness, apex thickness [4], teat canal [16,24], and milkability and udder health. 

Generally, the findings of studies focused on internal teat traits are not used in practice despite their proven strong relationship with udder health. The importance of introducing a working evaluation system based on objective measurements of individual traits combined with milkability parameters could help in their more comprehensive implementation into practice. The morphology of teat structures affects milking-induced changes; however, the extent of these changes might be influenced by milkability during milking. This study aimed to explore the relationship between milkability, the morphology of teat structures, and their milking-induced changes based on the hypotheses that teat structures affect milkability and, conversely, that milkability affects milking-induced changes. The goal was to create a model to evaluate the potential to predict the morphology of teat structures and their changes during milking based on milkability parameters. 

## 2. Materials and Methods

The study was conducted in accordance with the Czech legislation for the protection of animals against abuse (no. 246/1992) [25] and with the directive 2010/63/EU [26] on the protection of animals used for scientific purposes. The experimental protocol was approved by the scientific board of the Czech University of Life Sciences.

The study was conducted on a commercial dairy farm with Holstein cows in the Central Bohemian region of the Czech Republic (412 m above sea level). The cows were housed in a modern stable with free-stall housing with recycled manure solids as bedding. An electric cow brush was available in each section of the stable. Cows were milked twice daily in a herringbone parlor (2 × 12). The critical milk flow for the automatic detachment system was set at 0.5 kg.min^−1^. The pulsation was set at a 60:40 ratio, with 55 pulses per minute. The vacuum level was set at 42 kPa. The teat liners had a triangular design, with a 22.5 mm orifice diameter (Milkrite ImpulseAir IP10U ventilated; Milkrite InterPuls; Johnson Creek, WI, USA).

The morphological dimensions of the teat structures were measured from mid to late lactation (from 100 DIM to 314 DIM). Forty-eight Holstein cows (first lactation n = 14; second lactation n = 17; third lactation n = 10; fourth lactation n = 8) were evaluated.

Teat measurements were taken immediately before and after evening milking by a trained researcher. Teat length (LENGTH) was measured with a caliper. Teat barrel thickness (BARREL), teat cistern width (CISTERN), teat wall thickness (WALL), teat apex thickness (APEX), and teat canal length (CANAL) were measured by ultrasound (Aloka SSD500 machine with 7.5 MHz linear probe; Hitachi Aloka Medical; Tokyo, Japan), using the warm water bath method [1]. We chose to use the water bath method over the direct contact method because it provides better images of the whole teat without any possible deformations due to a lack of contact between the probe and the teat or udder tissue. The water in the cup was changed after each teat to avoid cross-contamination with mastitis pathogens. 

The collected ultrasound video recordings were processed in the NIS-Elements AR 3.2 (Nikon Corp.; Tokyo, Japan). Teat structures were measured at standard positions, as in the study by Neijenhuis et al. [20] or Wieland et al. [27]. Therefore, BARREL, CISTERN, and WALL were measured at 10 mm from Furstenberg’s rosette, while APEX was measured at Furstenberg’s rosette. CANAL was measured from Furstenberg’s rosette to the end of the teat. Milking-induced changes in LENGTH (LENGTH%), BARREL (BARREL%), CISTERN (CISTERN%), WALL (WALL%), APEX (APEX%), and CANAL (CANAL%) were calculated as:

Data for milk yield (milk yield in kg (MY); milk yield during the first two minutes of milking in kg (MY2 min)) and milkability (milking time in min (MT), average milk flow in kg.min^−1^ (AMF), partial milk flows 0–15 s, 15–30 s, 30–60 s, 60–120 s in kg.min^−1^ (MF0-15, MF15-30, MF30-60, MF60-120) were measured by in-line real-time milk analyzers (Afilab with Afifarm 3.08 software) [28] for each milking. Corresponding data with the time of ultrasonographic measurements were further evaluated. The occurrence of delayed milking was noted when there was no milk flow during the first 15 s of milking. The occurrence of bimodal milk flow was recorded if two increases in milk flow were followed by a significant decrease in milk flow by more than 0.2 kg.min^−1^ within 1 min after the start of milking [29]. We chose to use the milkings in the mid to late lactation based on our previous study [30], which concluded that changes early at the beginning of the lactation are more variable, whereas changes towards the end of lactation become more uniform. 

The SAS software ver. 9.4 [31] was used for statistical analyses. The basic statistics were calculated using the UNIVARIATE procedure. Correlation analysis between the monitored variables was performed by the CORR procedure. Multifactorial analysis of variance with the repeated effect of the animal was evaluated using the MIXED procedure. The evaluated fixed effects were divided into equal groups based on the standard deviation (group 1 = <−1/2 SD; group 2 = −1/2 SD to +1/2 SD; group 3 = >+1/2 SD). The REG procedure, the STEPWISE method, and the Akaike information criterion parameter were used to select appropriate effects for the model equation. Differences between means were evaluated using the Tukey–Kramer test. The first model equation for the evaluation of milkability parameters with teat morphology effects was as follows:

Y_ijklm_ = µ + TP_i_ + LO_j_ + MO_k_ + CW_l_ + e_ijklm_ (1)

where:

1. Y_ijkl_—measured value of the dependent variables (milk yield—MY [kg]; milk yield during the first two minutes of milking—MY2 min [kg]; milking time—MT [min]; average milk flow—AMF [kg.min^−1^]; partial milk flows 30–60 s—MF30-60 [kg.min^−1^]; the occurrence of delayed milk flow [%]; the occurrence of bimodal milk flow [%]);
2. µ—mean value of the dependent variable;
3. TP_i_—fixed effect group of teat parameters (LENGTH variant—i ≤ 43.5 µm), n = 162; i = 43.51–52.5 µm, n = 199; i ≥ 52.51 µm, n = 195; BARREL variant—i ≤ 24.2 µm, n = 210; i = 24.21–26.5 µm, n = 211; i ≥ 26.51 µm), n = 132; CISTERN variant—i ≤ 11 µm, n = 202; i = 11.01–14.5 µm, n = 201; i ≥ 14.51 µm, n = 153; WALL variant—i ≤ 5.7 µm), n = 102; i = 5.71–7 µm, n = 207; i ≥ 7.01 µm, n = 247; APEX variant—i ≤ 21 µm, n = 210; i = 21.01–22.5 µm, n = 180; i ≥ 22.51 µm, n = 164; CANAL variant—i ≤ 11.8 µm, n = 139; i = 11.81–14 µm, n = 222; i ≥ 14.01 µm, n = 195);
4. LO_j_—fixed effect of lactation order (j = 1, n = 164; j = 2, n = 180; j = 3 and subsequent, n = 212);
5. MO_k_—fixed effect month of evaluation (k = 1, n = 132; k = 5, n = 84; k = 6, n = 60; k = 11, n = 104; k = 12, n = 174);
6. CW_l_—fixed repeated effect of cows (48 cows in 4 to 16 repetitions = 4 teats x multiple measurements).
7. e_ijklm_—residual errors.

The second model equation for changes in teat morphology with milkability effects, was as follows:

Y_ijklm_ = µ + MA_i_ + LO_j_ + MO_k_ + CW_l_ + e_ijklm_ (2)

where:

1. Y_ijklm_—measured value of the dependent variables (change in LENGTH—LENGTH%; change in BARREL—BARREL%; change in CISTERN—CISTERN%; change in WALL—WALL%; change in APEX—APEX%; change in CANAL—CANAL%;
2. µ—mean value of the dependent variable;
3. MA_i_—fixed effect group of milkability parameters (milk yield variant—i ≤ 13 kg), n = 168; i = 13.01–16 kg, n = 216; i ≥ 16.01 µm, n = 172; milk yield during the first two minutes of milking variant—i ≤ 4.5 kg, n = 180; i 4.51–6 kg, n = 188; i ≥ 6.01 kg, n = 188; milking time variant—i ≤ 5 min., n = 112; i = 5.01–7 min., n = 288; i ≥ 7.01 min., n = 156; partial milk flow 30–60 s variant—i ≤ 2.5 kg.min^−1^, n = 160; i = 2.51–3.5 kg.min^−1^, n = 260; i ≥ 3.51 kg.min^−1^, n = 136; delayed flow variant—i = no, n = 164; i = yes, n = 392; average milk flow variant—i ≤ 2 kg.min^−1^, n = 128; i = 2.01–2.7 kg.min^−1^, n = 268; i ≥ 2.71 kg.min^−1^, n = 160; bimodal milk flow variant—i = yes, n = 404; i = no, n = 152);
4. LO_j_—lactation order fixed effect (j = 1, n = 164; j = 2, n = 180; j = 3 and subsequent, n = 212);
5. MO_k_—fixed effect month of evaluation (k = 1, n = 132; k = 5, n = 84; k = 6, n = 60; k = 11, n = 104; k = 12, n = 174);
6. CW_l_—fixed repeated effect of cows (48 cows in 4 to 16 repetitions = 4 teats x multiple measurements).
7. e_ijklm_—residual errors.

Finally, regression equations were calculated between selected milk ability parameters and teat morphology. Only statistically significant regression equations are presented in the results.

The significance levels for all evaluations were set at *p* < 0.05 and *p* < 0.01.

## 3. Results

### 3.1. Basic Statistics

The tested Holstein cows had a milk yield per milking of 14.7 kg with an average milking time of 6.3 min and an average milk flow of 2.4 kg/min for the milkings during the ultrasonographic measurements. The occurrence of bimodal milk flows was at 27.30%. The milk yield during the first two minutes of milking was 37.90% of the total milk yield, while the average milk flow between 0 and 15 s of milking was 0.3 kg/min; between 15 and 30 s was 2.67 kg/min; between 30 and 60 s was 2.94; and between 60 and 120 s was 3.28 kg/min.

Based on correlation analysis, morphology correlates with milkability more than milking-induced changes with milkability (Table 1). Most correlations with milkability parameters were observed for teat length (6 out of 9), apex (5 out of 9), and cistern (4 out of 9), whereas the teat wall only correlated with milking time (−0.107, *p* < 0.05). Relationships between milking-induced changes and milkability were rarely significant, and if so, they showed only weak relations. This might suggest non-linear or non-existent relations between milkability and morphology changes. On the other hand, correlations between morphology and their milking-induced changes were strong and significant (Table 2). 

### 3.2. Effects of Teat Morphology on Milkability

Teat morphology had a significant effect on milkability parameters (Table 3). Teat length was the only structure that did not affect milk yield. Small teats showed higher milk flow higher milk yield during the first two minutes, and a tendency for lower occurrence of bimodal flows. A significantly lower occurrence of bimodal flows was also observed for thick teats (17.34%, *p* < 0.05). Medium teats showed the best results for milkability with high milk yield and fast milking (Table 3). 

Teats with wide cisterns showed significantly higher milk yields and longer milking times. On the other hand, the wall showed the highest milk yield and longest milking time for thin walls. The least statistically significant differences in milkability parameters were observed for the APEX and WALL groups for milk yield and milking time (Table 3). Most milkability parameters were affected by CANAL groups, with significant differences noted between short and medium canals. Short canals showed higher milk yields, higher milk flows, and the lowest chance for delayed milking. 

Following our hypotheses, Table 4 shows the effect of milkability on milking-induced changes in teat structures. Milk yield had the most significant effect and affected all teat structures except teat wall changes. In general, higher milk yield caused more changes, whether negative or positive. Lower milk yields resulted in a reduced apex, while higher milk yields resulted in a slightly thickened apex. Milk yield during the first two minutes showed similar trends to milk yield. However, only teat and canal length were significantly affected, with greater changes noted for higher milk yields. 

Milking time affected the changes in BARREL, CISTERN, and WALL. Long milkings over 7 min caused a wall thickening of 28.85%. Although the wall thickened significantly, the barrel width decreased significantly by 6.10%. Partial milk flows, especially 30–60 s, had more significant effects on milking-induced changes than average milk flow, for which we did not observe any significant differences between groups. The tendency for higher milking-induced changes with higher milk flow was observed for teat canal length. 

On the other hand, the occurrence of delayed milking caused significantly smaller changes in teat canal length. The occurrence of delayed milking did not significantly affect changes in other teat structures. The presence of bimodal milk flows caused smaller changes in teat length and reduced changes in barrel thickness but did not affect changes in other structures (Table 4). 

As the results show, milkability factors have a variable effect on milking-induced changes in individual teat structures. Each change was affected by three or four milkability factors. Changes in CANAL were affected by four milkability factors: BARREL, LENGTH, and CISTERN were influenced by three factors (Table 4). We only observed significant differences between the WALL group for two milkability effects and only one for APEX. Therefore, we could estimate that if the milk yield were high, milk flow was fast, and the milking was not delayed, the changes in the teat canal would be 12 to 14%. If the milk yield was low, the milking time was less than 5 min, and bimodal flow occurred, then the changes in length would be in the lower range, the changes in the barrel would be closer to 0%, and the changes in cistern would be somewhere between −20 and −25%. 

### 3.3. Regression Equations

In the previous tables, we have shown significant relationships between milkability and morphology and milking-induced changes. Of course, not all structures have a significant effect on all milkability traits, and not all milkability traits have a significant effect on changes. However, there are strong relations between some of them, and choosing the correct ones could help to predict teat morphology and their milking-induced changes based on milkability.

As we can see in some examples below, the regressions equation showed some potential. The prediction of dimensions for LENGTH and CISTERN based on three milkability traits was significant but only explained 3.36% and 7.59% of the variability, respectively. The predictions for milking-induced changes were also significant and explained much higher variability (21.50% to 46.03%). However, due to the strong relationship between morphology and milking-induced changes, a structural dimension must be added to the regression equation. Therefore, changes cannot be predicted from milkability data alone. However, it might be possible to estimate teat structure dimensions based on milkability. Subsequently, we could estimate milking-induced changes based on teat morphology with added milkability traits, improving the accuracy of these predictions.

The examples of model equations to predict teat structure dimensions and their milking-induced changes based on milkability parameters are shown below:

1. LENGTH = 56.68 + AMF × (−4.579) + MF30-60 × (−2.592) + MY2 min × (2.060)
  R-square 3.36%; *p* < 0.001
2. CISTERN = 6.924 + MY × (−0.163) + MT × (0.819) + MF30-60 × (0.931)
  R-square 7.59%; *p* < 0.001
3. CANAL% = 59.210 + CANAL × (−4.720) + MT × (0.90) + MY2 min × (1.247)
  R-square 46.03%; *p* < 0.001
4. LENGTH% = 44.185 + LENGTH × (−0.654) + MT × (0.726)
  R-square 21.5%; *p* < 0.001

LENGTH—teat length (mm); CISTERN—teat cistern diameter (mm); CANAL—teat canal length (mm); MY2 min—milk yield during the first two minutes of milking; MF30-60—partial milk flows during 30–60 s of milking; AMF—average milk flow; MF3060—partial milk flows during 30–60 s of milking; MY—milk yield; MT—milking time

## 4. Discussion

The utilization of high-resolution ultrasonography in our trials showed that it could still be applied easily and without stress to the animals due to the non-invasive nature of the technique. Accurate measurements were made possible by a high image quality and were based on the implementation of highly accurate measurement software. The analysis of all images by the same observer is emphasized by Wieland et al. [21], who mention the risk of reduced precision of estimates and the ability to detect correlations of interest when measurements are performed by various observers. However, teat ultrasonography measurements would be difficult to implement in daily practice. It is impossible to manually scan every cow in the herd every day due to the time-consuming scanning and interpretation process. Therefore, there is a need to develop an effective method that would help to estimate changes in internal structures, e.g., by using milkability parameters. 

We hypothesized that teat morphology would influence milkability and that milkability would subsequently influence milking-induced changes. Although the relationship between the dimensions of teat structures and their changes during milking is very strong [12,20,21,30], our results showed that milkability parameters also have a significant effect on the extent of these changes. Milkability parameters could be used to predict the dimensions of teat structures and their milking-induced changes. However, this potential is limited. The predictive ability improves dramatically when initial dimensions are provided. Changes in structures during milking influence the development of teat structures over the production life [4,16,20,21]. If teat dimensions were measured at the beginning of the production life, automatically collected milkability data could be used to calculate milking-induced changes for each milking and how they would affect the development of teat dimensions over the production life.

We found that some structures were significantly affected by more milkability parameters than others, with the teat canal and its change being affected by the most milkability parameters. Many studies agree that teat canal length has a significant effect on the flow of milk through the teat canal [12]. We found that short canals had higher milk yields, higher milk flows, and the lowest chance of delayed milking. These findings are consistent with other studies, where the teat canal length had a significant effect on milk flow characteristics such as daily milk yield, average milk flow, maximum milk flow, and somatic cell score [1,13,16,32]. Teat canal length, as well as other teat canal morphological characteristics, may influence the development of udder infections [1]. In addition, high teat canal changes were found to be a sign of milk flow disturbances [33]; however, we observed higher CANAL% with desirable milkability parameters. The teat canal is considered to be the cow’s primary defense against mastitis pathogens [34]. Therefore, resistance to intramammary infections is critical to ensure the integrity of the teat canal and the pliability of the adjacent tissues [14,34]. Klein et al. [24] recommend long and narrow teats to improve udder health. They also found that a greater teat canal diameter was associated with a higher incidence of mastitis. Therefore, predicting canal length and its changes during milking over the production life could provide valuable information to farm managers, who could be alerted about risks for udder health for specific cows. 

Milking-induced changes in barrel thickness have been reported to be associated with udder health [8]. Our study found that BARREL% was significantly affected by different milk yield, bimodal flows, and milking time groups. We also found that the highest decrease in BARREL% occurred when the milkability factors applied caused severe thickening of WALL%. Therefore, the effects of milkability on BARREL% and WALL% were indirectly proportional. Long milking times of more than 7 min caused the thickening of the wall by 28.85%, while 5 to 7 min of milking caused significantly less wall thickening (21.97%; *p* < 0.05). Even though the wall thickened significantly, barrel width significantly decreased by 6.10%. Long milkings are often associated with longer periods of overmilking, which has been shown to cause higher WALL% and lower BARREL% [9] and are known to damage the teat tissue and cause hyperkeratosis [35]. Reduced intramammary pressure and muscle tone [20] and elongation of the teat may account for a decrease in teat diameter after milking, whereas accumulation of fluid in the teat wall results in positive changes in teat wall thickness [20,36]. Congestion or edema can impair blood circulation and thus reduce the efficiency of teat defense mechanisms such as neutrophil activity [20], which may explain the increased SCC with increasing positive diameter changes at the teat barrel [8]. However, we did not observe positive BARREL% in any group, whereas WALL% thickened significantly under certain milkability conditions. 

Teat length correlated with most of the milkability parameters of the observed structures and showed a significant effect on three milkability parameters, and LENGTH% was significantly influenced by three milkability parameters. The importance of uniform teat length is well known and has long been a part of selection indexes for udder conformation. Teat length is mainly important because of its close interaction with the teat liner, as teats that are too short or too long may not fit properly into the liner and may not be massaged efficiently [16]. 

Variation in teat dimensions during lactation may be determined by a combination of factors. Teat length and diameter may be associated with milk yield, as suggested by several studies [20,21]. We found that small teats had higher milk flow, higher milk yield during the first two minutes and a tendency for lower occurrence of bimodal flows. A significantly lower occurrence of bimodal flows was also observed for thick teats (17.34%, *p* < 0.05). Medium teats showed the best results for milkability with high milk yield and fast milking. Similar findings for teat length were confirmed by Weiss et al. [16], where teat length was negatively related to peak flow rate and average flow rate at the udder level. Furthermore, the action of the milking machine may cause long-term changes in teat dimensions.

Other structures were less affected, and we only found a significant relation to one or two milkability parameters. APEX%, which has been shown to be an important indicator of udder health and effective milking [14], was only significantly affected by milk yield. On the other hand, there is a strong relation between APEX% (milking-induced change in teat apex thickness) and APEX [37], and APEX significantly affected milk yield and milking time. APEX has been found to be related to udder health in studies [4], which found an increased chance for mastitis by 20%, with each increase in the pre-milking diameter of APEX by 1 mm, and the authors attributed it to wider teat canals and larger orifices in wider teats. Furthermore, changes in teat apex diameter of more than 5% increased pathogen colonization of the teat canal [22]. A similar threshold of ±5% change in apex diameter was suggested by Hamann and Mein [10] to evaluate the effectiveness of pulsation in relation to the liner type and vacuum level. Due to the significance of APEX and APEX% for udder health and milking efficiency (edema formation, hyperkeratosis), it would be important to predict their changes during milking and over the production life. Teat liner widths and milking settings, such as vacuum and pulsation, were found to affect APEX and APEX% [38,39]. Including these effects together with the initial dimensions at the beginning of a cow’s production life may improve the accuracy of the prediction, but studies would be needed. 

As mentioned above, many studies have indicated that teat structures should be continuously monitored throughout the production life of dairy cows to protect their udder health. Several other factors have also been identified and associated with the quality of the team’s defense mechanisms, such as udder depth, fore udder attachment, teat length, teat shape, and milkability [40]. Relationships between teat shape and measurements with milk yield and other milkability parameters should be regarded as criteria for the selection of dairy cattle [41]. Nørstebø et al. [17] concluded that information on milk flow rate is readily available in automated milking systems and that this information could be used to evaluate the causes of impaired teat-end condition.

Our findings suggest that some teat structures could be reliably predicted based on these milkability parameters. For example, we could estimate that if the milk yield were high, milk flow was fast, and the milking was not delayed, teat canal changes would be 12 to 14%. If the milk yield was low, the milking time was less than 5 min, with an occurrence of bimodal flow, then the changes in length would be in the lower range, the changes in the barrel would be closer to 0%, and changes in cistern would be somewhere between −20 and −25%. Furthermore, this prediction could be improved by including factors that significantly affect teat structures, such as milking settings, teat liner, and breed. If improved, this modeling could be a useful tool for farm managers and companies that develop and sell milking equipment. This tool could provide alerts about critical milking-induced changes and risky teat conformation traits, as teat structures and their milking-induced changes have been shown to affect udder health and are reliable indicators of milking efficiency.

## 5. Conclusions

Currently, when precision agriculture is increasingly being promoted in dairy herds and modern computer systems and procedures are being applied, it is very important to develop new options and automated solutions to improve the management of dairy farms. In this study, we have been able to test models that demonstrate the potential of milkability data to predict milking-induced changes and the dimensions of some teat structures. It may be possible to model the development of teat structures during production life based on milkability parameters, but more factors that may influence teat morphology and milking-induced changes need to be included to accurately predict all teat structures. A tool like this could be used to estimate milking-induced changes and alert farm managers to changes that could damage teats and negatively affect udder health. Our results and the results of other studies in this area of research could help to create a detailed computer model of the reaction to milking, which could also be used in the development of milking equipment or breeding for internal teat morphology traits.

## Figures and Tables

**Table 1:** Correlations of morphology and milking-induced changes in teat structures to milk yield and milkability represented by correlation coefficient.

	Milk Yield	MY2 min	Milking Time	MF0-15	MF15-30	MF30-60	MF60-120	AMF	Bimodal Flow
**LENGTH**	−0.019	−0.107 *	0.136 *	−0.086 *	−0.079	−0.132 *	−0.084 *	−0.143 *	−0.004
**BARREL**	0.123 *	0.016	0.153 *	−0.042	0.011	0.079	−0.010	−0.037	−0.135 *
**CISTERN**	0.109 *	0.042	0.172 *	0.003	0.021	0.094 *	0.019	−0.030	−0.090 *
**WALL**	0.003	0.035	−0.107 *	−0.030	0.045	0.028	0.039	0.075	0.045
**APEX**	0.129 *	0.036	0.136 *	−0.115 *	0.086 *	0.096 *	0.003	−0.029	−0.051
**CANAL**	0.115 *	0.027	0.022	−0.147 *	0.023	−0.012	0.066	0.036	0.063
**LENGTH%**	0.002	0.030	0.016	0.011	0.051	0.035	0.018	−0.002	0.065
**BARREL%**	−0.053	0.010	−0.073	−0.003	0.055	−0.028	0.017	0.031	0.107 *
**CISTERN%**	−0.082 *	−0.028	−0.095 *	−0.007	−0.039	−0.048	−0.009	0.013	0.028
**WALL%**	0.023	0.063	0.020	0.088 *	0.021	0.085 *	0.049	0.037	−0.065
**APEX%**	0.047	−0.005	−0.006	0.043	−0.050	−0.022	0.014	0.044	−0.002
**CANAL%**	0.028	0.077	0.001	0.083 *	0.050	0.100 *	0.057	0.023	−0.084 *

* means statistically significant *p* < 0.05. MY2 min—milk yield during the first two minutes of milking; MF0-15—partial milk flow during 0–15 s of milking; MF15-30—partial milk flow during 15–30 s of milking; MF30-60—partial milk flow during 30–60 s; MF60-120—partial milk flow during 60–120 s of milking; AMF—average milk flow; bimodal flow—the occurrence of bimodal milk flow; LENGTH—teat length; BARREL—teat barrel diameter; CISTERN—teat cistern diameter; WALL—teat wall diameter; APEX—teat apex diameter; CANAL—teat canal length; LENGTH%—change in teat length; BARREL%—change in teat barrel diameter; CISTERN%—change in teat cistern diameter; WALL%—change in teat wall diameter; APEX%—change in teat apex diameter; CANAL%—change in teat canal length.

**Table 2:** Correlations between dimensions of teat structures and their milking-induced changes represented by correlation coefficient.

	LENGTH%	BARREL%	CISTERN%	WALL%	APEX%	CANAL%
**LENGTH**	−0.457 *	−0.084 *	−0.012	−0.069	−0.067	0.029
**BARREL**	−0.044	−0.602 *	−0.345 *	0.134 *	−0.155 *	0.126 *
**CISTERN**	−0.059	−0.436 *	−0.520 *	0.338 *	−0.209 *	0.054
**WALL**	0.024	0.193 *	0.309 *	−0.558 *	0.270 *	0.050
**APEX**	−0.018	−0.236 *	−0.131 *	0.094 *	−0.542 *	0.035
**CANAL**	−0.144 *	0.184 *	0.255 *	−0.307 *	−0.023	−0.047

* means statistically significant *p* < 0.05. LENGTH%—change in teat length; BARREL%—change in teat barrel diameter; CISTERN%—change in teat cistern diameter; WALL%—change in teat wall diameter; APEX%—change in teat apex diameter; CANAL%—change in teat canal length; LENGTH—teat length; BARREL—teat barrel diameter; CISTERN—teat cistern diameter; WALL—teat wall diameter; APEX—teat apex diameter; CANAL—teat canal length.

**Table 3:** The effect of teat structures’ dimensions on monitored milkability parameters.

Effect	Groups	Milk Yield (kg)	MY2 min (kg)	Milking Time (min)	MF30-60 (kg.min^−1^)	AMF (kg.min^−1^)	Delayed Flow (%)	Bimodal Flow (%)
**LENGTH** (mm)	<43.5	14.70	5.84 ^b^	5.95	3.16 ^A, a^	2.52 ^A^	61.17	21.68
	43.5–52.5	14.31	5.36 ^a^	6.18	2.85 ^b^	2.37 ^B^	70.76	28.66
	>52.5	14.20	5.39 ^a^	6.26	2.82 ^B^	2.36 ^B^	67.07	27.83
**BARREL** (mm)	<24.2	13.89 ^A, a^	5.32	5.96 ^A^	2.75 ^a^	2.39	71.13	30.37 ^a^
	24.2–26.5	14.50 ^b^	5.59	6.08 ^a^	2.99	2.44	65.45	28.60 ^a^
	>26.5	14.91 ^B^	5.66	6.49 ^B, b^	3.09 ^b^	2.40	63.08	17.34 ^b^
**CISTERN** (mm)	<11	13.89 ^A^	5.35	5.95 ^A^	2.78 ^a^	2.38	67.39	30.47
	11–14.5	14.39	5.53	6.03 ^A^	2.98	2.44	68.79	24.34
	>14.5	15.00 ^B^	5.69	6.50 ^B^	3.07 ^b^	2.42	62.82	23.68
**WALL** (mm)	<5.7	14.86 ^a^	5.53	6.48 ^A^	2.98	2.38	62.89	23.89
	5.7–7	14.42	5.50	6.18	2.89	2.41	66.99	26.09
	>7	14.12 ^b^	5.52	5.93 ^B^	2.95	2.43	68.13	27.68
**APEX** (mm)	<21	13.82 ^A^	5.44	5.87 ^A^	2.80	2.42	63.20	30.34
	21–22.5	14.70 ^B^	5.57	6.17	3.00	2.44	64.71	24.32
	>22.5	14.77 ^B^	5.55	6.43 ^B^	3.04	2.39	72.72	22.95
**CANAL** (mm)	<11.8	15.08 ^A^	5.80 ^a^	6.27	3.16 ^a^	2.51 ^a^	55.67 ^a^	24.70
	11.8–14	13.90 ^B^	5.31 ^b^	6.11	2.79 ^b^	2.34 ^b^	70.06 ^b^	24.23
	>14	14.40	5.51	6.07	2.92	2.43	71.01 ^b^	29.65

Different letters in columns within effect indicate statistically significant differences A,B *p* < 0.01; a,b *p* < 0.05. MY2 min—milk yield during first the two minutes of milking; MF30-60—partial milk flow during 30–60 s of milking; AMF—average milk flow; delayed flow—the occurrence of delayed milk flow; bimodal flow—the occurrence of bimodal milk flow; LENGTH—teat length; BARREL—teat barrel diameter; CISTERN—teat cistern diameter; WALL—teat wall diameter; APEX—teat apex diameter; CANAL—teat canal length.

**Table 4:** The effect of milk yield and milkability on milking-induced changes in teat structures.

Effect	Groups	LENGTH%	BARREL%	CISTERN%	WALL%	APEX%	CANAL%
**Milk yield (kg)**	<13	14.13 ^a^	−4.04 ^a^	−23.92 ^A^	21.10	−0.88 ^a^	7.48 ^A^
	13–16	16.29	−5.99 ^b^	−27.81	26.27	−0.07	10.33
	>16	18.46 ^b^	−5.22	−33.90 ^B, b^	24.43	1.06 ^b^	12.57 ^B^
**MY2 min (kg)**	< 4.5	14.13 ^a^	−4.27	−26.13	21.57	−0.12	8.07 ^A^
	4.5–6	16.29	−5.81	−28.20	24.38	0.09	8.32 ^A^
	>6	18.46 ^b^	−5.15	−29.39	25.94	−0.21	13.35 ^B^
**MY2 min (%)**	<30	14.93	−5.12	−29.09	26.23	0.24	10.35
	30–45	16.89	−5.80 ^a^	−28.36	22.09	−0.01	8.66
	>45	15.67	−3.99 ^b^	−26.29	25.40	−0.45	11.58
**Milking time (min)**	<5	15.97	−3.57 ^a^	−21.97 ^A, a^	23.59	−0.33	10.36
	5–7	15.81	−5.32	−28.55 ^b^	21.90 ^A^	−0.16	9.19
	>7	16.72	−6.09 ^b^	−31.97 ^B^	28.85 ^B^	0.32	10.94
**MF30-60 (kg.min** **^−1^)**	<2.5	15.07	−4.44	−24.35 ^a^	19.04 ^A^. ^a^	0.12	7.52 ^A^
	2.5–3.5	16.29	−5.64	−29.51 ^b^	26.14 ^B^	−0.08	9.39 ^a^
	>3.5	16.78	−4.85	−28.88	25.44 ^b^	−0.29	13.70 ^B, b^
**Delayed flow**	no	16.62	−5.27	−27.98	25.92	0.20	11.82 ^a^
	yes	15.79	−5.06	−27.94	23.08	−0.21	8.92 ^b^
**Average milk flow (kg.min** **^−1^)**	<2	15.56	−4.91	−26.44	22.01	0.12	8.82
	2–2.7	15.74	−5.24	−28.80	25.09	−0.32	9.15
	>2.7	16.99	−5.13	−27.87	24.03	0.15	11.87
**Bimodal flow**	yes	15.21 ^A^	−5.54 ^a^	−28.22	24.91	−0.02	10.50
	no	18.48 ^B^	−3.96 ^b^	−27.22	21.57	−0.23	8.18

Different letters in columns within effect represent statistically significant differences A,B *p* < 0.01; a,b *p* < 0.05; LENGTH%—change in teat length; BARREL%—change in teat barrel diameter; CISTERN%—change in teat cistern diameter; WALL%—change in teat wall diameter; APEX%—change in teat apex diameter; CANAL%—change in teat canal length; MY2 min—milk yield during first two minutes of milking; MF3060—partial milk flows during 30–60 s of milking; AMF—average milk flow; delayed flow—the occurrence of delayed milk flow; bimodal flow—the occurrence of bimodal milk flow.

## Data Availability

The original contributions presented in the study are included in the article. Further inquiries can be directed to the corresponding author/s.
